# Poorly cohesive duodenal carcinoma mixed with signet ring cell carcinoma with systemic metastasis: a case report and literature review

**DOI:** 10.3389/fonc.2023.1240013

**Published:** 2023-08-24

**Authors:** Song Tang, Xinjun Li, Aiguo Wu

**Affiliations:** ^1^ Department of Oncological Surgery, Guangzhou Royallee Cancer Center, Guangzhou, China; ^2^ Department of General Surgery, Zhujiang Hospital of Southern Medical University, Guangzhou, China

**Keywords:** duodenal carcinoma, poorly cohesive carcinoma, signet ring cell carcinoma, systemic metastasis, case report

## Abstract

Poorly cohesive duodenal carcinoma mixed with signet ring cell carcinoma is very rare, and no cases have been reported. When distant metastasis occurs, it is very easy to be misdiagnosed. We report the first case of a 52-year-old man with poorly cohesive carcinoma of the duodenum mixed with signet ring cell carcinoma with systemic metastasis. The process of its diagnosis and differential diagnosis is highlighted.

## Introduction

Primary duodenal cancer is rare, accounting for only 0.3%–0.5% of cancers of the digestive tract (1). Duodenal adenocarcinoma is the most common type of duodenal cancer. In a study of duodenal cancer, the proportion was subdivided into adenocarcinoma 87%, mucinous adenocarcinoma 7%, and signet ring cell carcinoma 1% (2). According to the fifth edition of the WHO Classification of Tumors of the Digestive System (3), among tumors of the small intestine and ampullary region, poorly cohesive carcinoma (PCC) is classified as adenocarcinoma of the small intestine not otherwise specified (SBAs-NOS). Among the subtypes of poorly cohesive small intestinal carcinomas, PCC not otherwise specified (PCC-NOS) consisting of less than 10% signet ring cells accounts for the majority, while PCC-NOS and signet ring cell carcinomas with 10%–90% signet ring cell components are relatively rare (4, 5). More than 96% of signet ring cell cancers occur in the stomach, with the remainder occurring primarily in the breast, gallbladder, pancreas, bladder, and intestine (6, 7).

Duodenal carcinoma is relatively rare, and poorly cohesive duodenal carcinoma mixed with signet ring cell carcinoma with systemic metastasis is very rare. It has not been fully elucidated in many aspects and is easy to be misdiagnosed. Therefore, this article will summarize the diagnosis and treatment process of poorly cohesive duodenal carcinoma mixed with signet ring cell carcinoma, so as to let clinicians have more understanding of the disease.

## Case presentation

A 52-year-old man had persistent cough for 1 month due to novel coronavirus infection, and the anticough drugs were not effective. Chest computed tomography (CT) performed at another hospital suggested the possibility of left lung cancer, with enlarged lymph nodes in both lungs and mediastinum. He came to our hospital for further treatment. Physical examination on admission revealed stable respiratory rate, regular rhythm, normal and symmetrical auscultation breath sounds, and no obvious rales and wheezing. Scattered patchy skin macules were seen on the trunk, chest, abdomen, back, and lower limbs. Multiple lymph nodes were palpable on the left supraclavicular and bilaterally in the groin.

**Figure 1:**
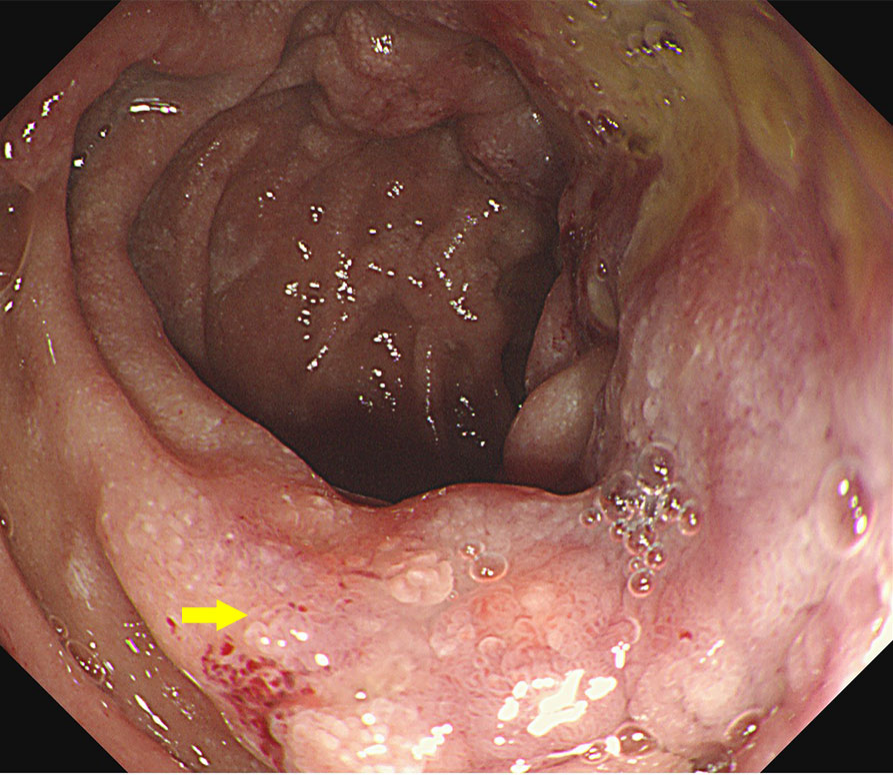
Endoscopic images at the time of the first admission. The descending duodenum was seen with thickening of the intestinal wall and surrounding ulceration.

**Figure 2:**
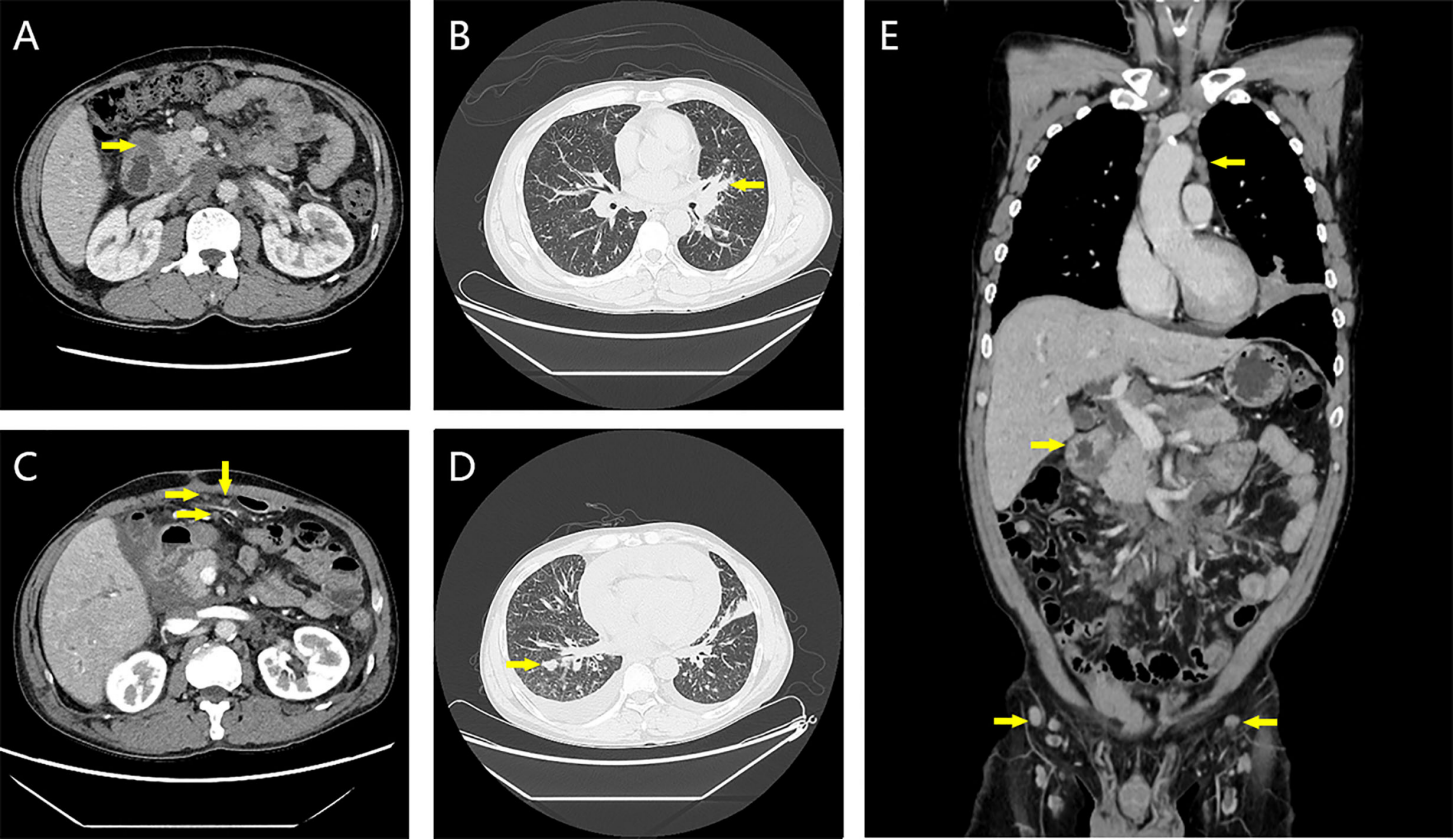
Contrast-enhanced CT images at first admission and 2 weeks after three cycles of chemotherapy. **(A)** The wall of the descending duodenum was thickened at the first admission. **(B)** Bronchus occlusion in the lingual segment of the left upper lobe of the lung on the first admission. **(C)** Irregular thickening of partial peritoneum, omentum, and mesentery at 2 weeks after the third cycle of chemotherapy. **(D)** New solid nodule in the right lung 2 weeks after the third cycle of chemotherapy. **(E)** On the first admission, thickening of the intestinal wall of the descending duodenum and multiple mediastinal and inguinal lymphadenopathy were seen.

After the patient’s admission, contrast-enhanced CT of the chest and abdomen, tumor markers for lung cancer, tumor markers for gastrointestinal tract, and gastrointestinal endoscopy were performed to further confirm the situation. Gastroscopy showed a mass in the descending duodenum (**Figure 1**). Contrast-enhanced CT of the chest and abdomen (**Figure 2**) was considered to be lymphoma, and the possibility of sarcoidosis and lung cancer could not be excluded. The specific findings were as follows: multiple lymph nodes in the left clavicle region, mediastinum, bilateral hilar lung, diaphragmatic crus, portal vein space, abdominal cavity, retroperitoneal cavity and bilateral groin, partial enlargement. The submucosal wall of the descending duodenum was scattered and thickened. Lymphoma was considered in all these cases, but sarcoidosis was not excluded. Bronchoscopic biopsy was performed to exclude lung cancer if necessary.

Multiple tumor markers were elevated: carcinoembryonic antigen (CEA), 2,687 ng/mL; neuron-specific enolase (NSE), 29.58 ng/mL; squamous cell carcinoma antigen (SCC), 6.2 ng/mL; cytokeratin 19 fragment assay (CYFRA21-1), 37.49 ng/mL; carbohydrate antigen (CA)125, 43.15 U/mL; CA72-4, 600 U/mL; CA19-9, 472.4 U/mL. See **Table 1** for the complete spelling of the abbreviations

**Table 1:** List of abbreviations for tumor markers and immunohistochemistry.

Abbreviations	Complete spelling
CEA	carcinoembryonic antigen
NSE	neuron-specific enolase
SCC	squamous cell carcinoma antigen
CYFRA21-1	cytokeratin 19 fragment assay
CA125	carbohydrate antigen 125
CA72-4	carbohydrate antigen 72-4
CA19-9	carbohydrate antigen 19-9
CK	cytokeratin
IMP-3	insulin-like growth factor 2 mRNA-binding protein 3
AB-PAS	alcian blue and periodic acid-Schiff
CDX2	caudal-type homeobox transcription factor 2
MUC	mucin
MUC5AC	mucin 5 Subtype AC
SATB2	special AT-rich sequence-binding protein 2
TTF-1	thyroid transcription factor 1
Napsin A	aspartic proteinase A

Because of the uncertainty of the diagnosis, a multidisciplinary consultation was conducted, including input from medical oncology, imaging, intervention, endoscopy, and intensive care units. First, fiberoptic bronchoscopy was performed to obtain tissue biopsy of the left upper bronchus, and puncture biopsy of the left lung mass was performed to determine the condition of the left lung mass. Second, biopsy of the left supraclavicular lymph node was performed. Third, biopsy of duodenal mass was taken under a gastroscope to confirm the situation. Fourth, positron emission tomography (PET) and computed tomography (CT) were performed to confirm the general condition.

PET-CT was the first to show the results. The extent of the tumor and lymph node was the same as that of CT, and lymphoma was also considered.

**Figure 3:**
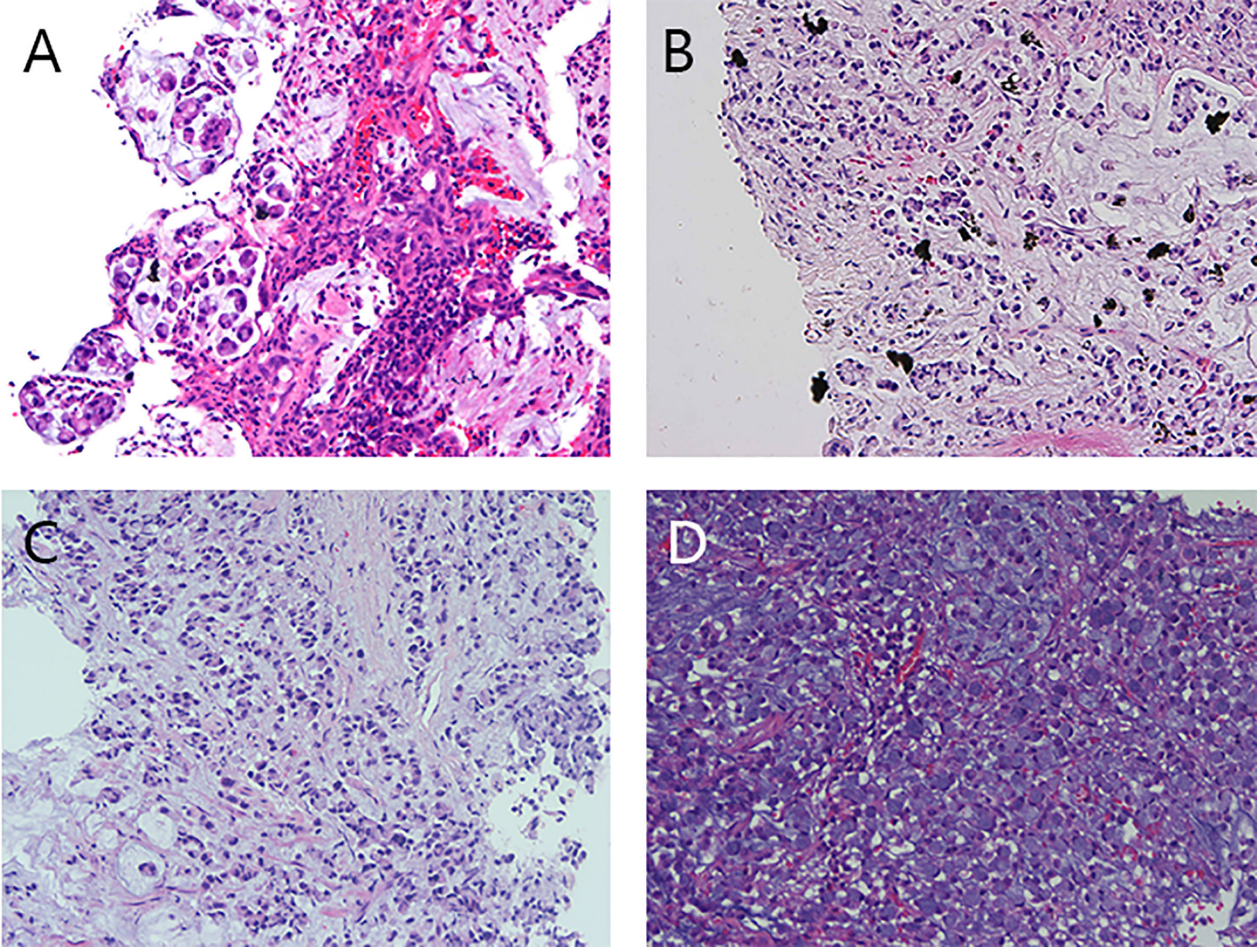
Preoperative pathological images of the descending duodenal mass, left lung mass, left upper bronchus, and left supraclavicular lymph node. **(A)** The pathological image of the mass in the descending duodenum showed mucus and a small amount of signet ring cells. **(B)** Pathological image of the left lung mass, showing mucus and signet ring cell mass. **(C)** Pathological image of the left upper bronchus, poorly cohesive carcinoma. **(D)** Left supraclavicular lymph node, lymph node cancer metastasis, poorly cohesive carcinoma was considered.

The biopsy results (**Figure 3**) indicate the presence of PCC in various locations: ①Descending duodenum: Mucus and a few signet ring cells were observed, consistent with PCC (including signet ring cell carcinoma). ②Left upper bronchus: Diagnosed as PCC. Combined with immunohistochemistry, the possibility of gastrointestinal metastasis was considered. ③Left lung mass: Presence of mucus and signet ring cell mass. Combined with immunohistochemical phenotype and clinical history, the tumor was consistent with lung metastasis of PCC of the digestive tract (including signet ring cell carcinoma). ④Left supraclavicular lymph node: Considered to be metastatic with poorly cohesive cancer.

**Figure 4:**
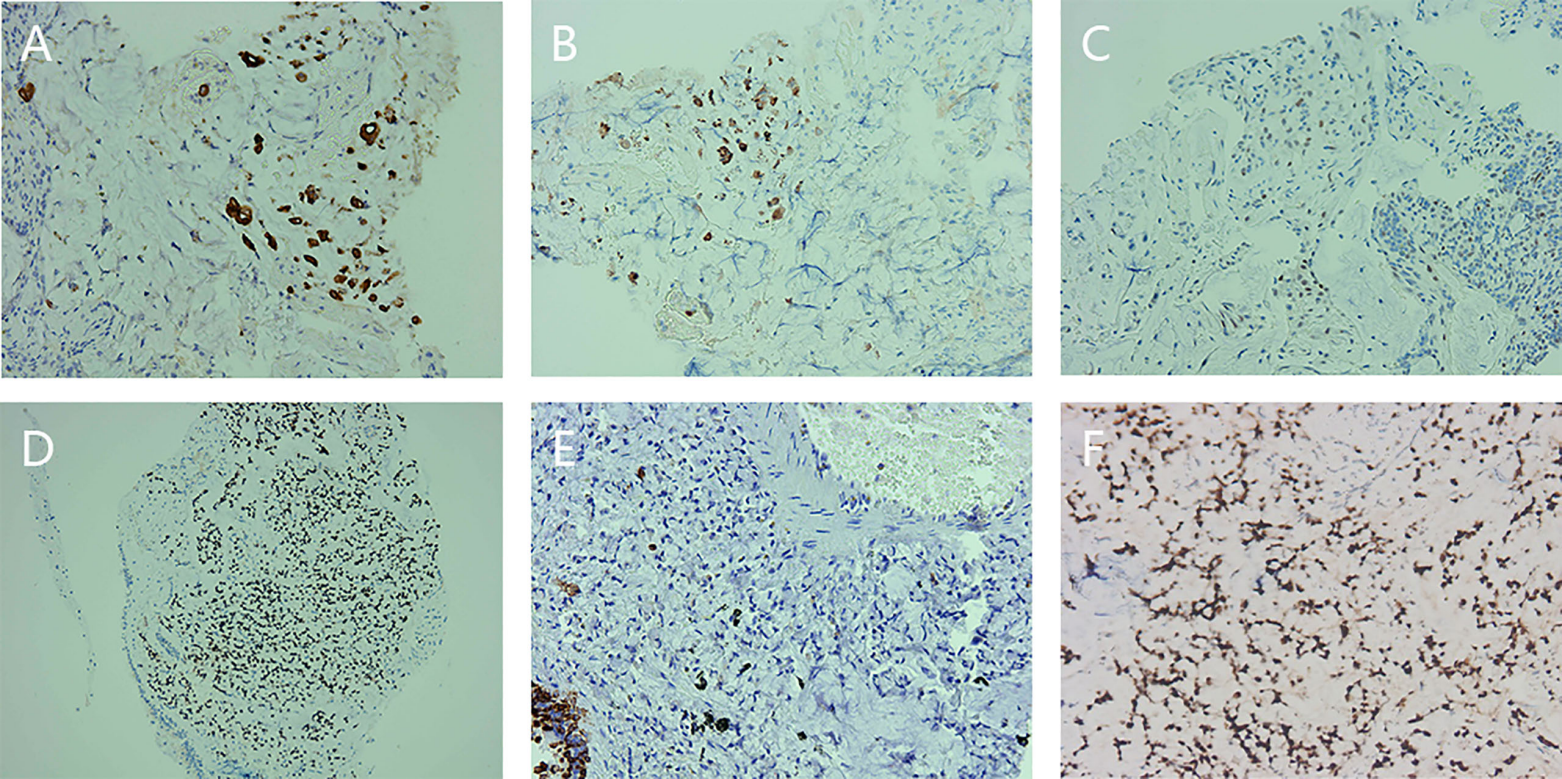
Preoperative immunohistochemical staining of descending duodenum, left lung mass, and left upper bronchus. **(A)** Immunohistochemical staining for cytokeratin (CK) expression in the descending duodenum. **(B)** Immunohistochemical staining for CK20 expression in the descending duodenum. **(C)** Immunohistochemical staining of P53 expression in the descending duodenum. **(D)** Immunohistochemical staining of caudal-type homeobox transcription factor 2 (CDX2) expression in the left lung mass. **(E)** Immunohistochemical staining of CK7 expression in the left lung mass. **(F)** Immunohistochemical staining for CDX2 expression in the left upper bronchus.

Immunohistochemical results were shown in **Figure 4** and **Table 2**.

**Table 2:** Preoperative immunohistochemistry.

	CK	CK7	CK20	KI-67	P53	IMP-3	AB- PAS	CDX2	MUC5 AC	MUC2	MUC4	SATB2	TTF-1	Napsin A
Descending duodenum	+	/	+	+(70%)	+	-	+	/	/	/	/	/	/	/
Left upper bronchus	+	+	+	+(90%)	+	/	/	+	+	/	+	+	+	-
Left lung mass	+	-	+	+(95%)	/	/	/	+	/	-	/	+	-	-

Based on the imaging and pathological and immunohistochemical results, the diagnosis of poorly cohesive duodenal carcinoma mixed with signet ring cell carcinoma with systemic metastasis was confirmed.

Considering the late stage and no possibility of radical resection, the patient and his family decided to undergo palliative bypass surgery to prevent further ampullary and duodenal obstruction. The specific plan was choledochojejunostomy, cholecystectomy, and Roux-en-y gastrojejunostomy.

The patient received three cycles of mFOLFOX6 (oxaliplatin 85 mg/m^2^, calcium folinate 400 mg/m^2^, fluorouracil 400 mg/m^2^, fluorouracil 2,400 mg/m^2^) chemotherapy after surgery. During this period, the patient’s left lung metastases and some lymph nodes shrank, and his cough resolved. The tumor progressed 2 weeks after cycle 3, with chest tightness, shortness of breath, cough, and expectoration. CT showed the possibility of bronchial spread, which was slightly progressive. The systemic lymph nodes were enlarged and increased, part of the peritoneum, omentum, and mesentery were irregularly thickened, implantation metastasis was considered, the bilateral pleurae were irregularly thickened, metastasis could not be excluded, and bilateral pleural effusion and pericardial effusion were massive. After supportive care, the patient received two cycles of FOLFIRI (irinotecan 180 mg/m^2^, calcium folinate 400 mg/m^2^, fluorouracil 400 mg/m^2^, fluorouracil 2,400 mg/m^2^) chemotherapy. The multidisciplinary consultation suggested that a large panel gene test should be performed to provide the basis for the next medication, but the patient with poor economic ability and short life expectancy refused our suggestion. Unfortunately, the tumor progressed rapidly, and the patient died of heart failure 35 days after cycle 2 of the FOLFIRI chemotherapy regimen.

## Discussion

Duodenal adenocarcinoma is the most common pathological type of duodenal cancer. It is most commonly found in the second part of the duodenum, followed by the third and fourth parts, and the first part, especially the duodenal bulb, is very rare (8–10). Our case of tumor was in the second part.

The pathological feature of PCC is that the PCC pattern accounts for more than 50% of the tumor (4). There are three subtypes of PCC in the small intestine. If the signet ring cells account for more than 90%, it is classified as signet ring cell carcinoma. If the signet ring cell component accounts for 10%–90%, it is classified as PCC and signet ring cell carcinoma not otherwise specified. Most PCCs in the small intestine consist of less than 10% signet ring cells, which are designated as PCC-NOS (4, 5). In our case, radical resection was not performed, only biopsy results were available, and the specific proportion of signet ring cell components was unknown, so it was difficult to accurately classify. However, combined with multiple pathological and immunohistochemical results, and the advice of pathologists, we considered the diagnosis of PCC mixed with signet ring cell carcinoma, that is, the proportion of signet ring cell component is 10%–90%.

Duodenal adenocarcinoma is usually difficult to diagnose. When symptoms appear, most of them are at an advanced stage, and the opportunity for radical resection is lost. Abdominal pain is the most common symptom of duodenal adenocarcinoma; other symptoms include nausea, vomiting, fatigue, weakness, and weight loss (8). Anemia, intestinal obstruction, and jaundice are advanced symptoms (8). However, the present case presented with cough as the first symptom, elevated amylase but no jaundice, and no abdominal pain so far, which undoubtedly increases the difficulty of diagnosis.

In terms of diagnosis and differential diagnosis, this case was mainly differentiated from lung cancer, lymphoma, sarcoidosis, and ampullary carcinoma in the actual process. In the presence of the possibility of multiple diseases, we first consider that all lesions and symptoms are caused by a single disease. Therefore, differential diagnosis is very important.

Endoscopy is the examination of choice for duodenal cancer and enables simultaneous visualization and biopsy (7, 8). Enhanced CT and magnetic resonance imaging (MRI) can be used to assess the depth of tumor invasion, regional lymph node invasion, and distant metastasis, which are important for determining tumor resectability as well as for treatment planning (7, 8, 11). PET and CT combined (PET-CT) have become important advanced imaging techniques in oncology, which are used in tumor staging, treatment response assessment, restaging, and longitudinal recurrence monitoring (12). They are also the most sensitive and accurate methods for staging distant metastasis (13). In this case, lung cancer, lymphoma, and sarcoidosis had been considered on imaging examinations. However, endoscopic biopsy and immunohistochemistry finally confirmed PCC mixed with signet ring cell carcinoma of the duodenum.

However, for duodenal carcinoma with distant metastasis, identification of the primary lesion and differential diagnosis require more examination methods, such as pathology, immunohistochemistry, and tumor markers. Thyroid transcription factor 1 (TTF-1) is a key single marker for lung adenocarcinoma (14). TTF-1, cytokeratin (CK)7, and CK20 can be used to distinguish primary and secondary lung adenocarcinomas, and the combination of TTF-1 negative, CK7 negative, and CK20 positive is highly correlated with adenocarcinoma of digestive tract origin (15). The immunohistochemistry of the left lung mass in our case belonged to this combination, so the primary tumor was considered to be of gastrointestinal origin.

Both duodenal carcinoma and ampullary carcinoma belong to periampullary carcinoma. It is often difficult to distinguish them based on morphology alone, but immunohistochemistry can help us to identify them. CK17, mucin 4 (MUC4), and MUC1 have been found to be expressed in the pancreaticobiliary type, whereas CK20, MUC2, and caudal-type homeobox transcription factor 2 (CDX2) are expressed in the intestinal type (16). In our case, CK20 and CDX2 were positive in the descending duodenum, left upper bronchus, and left lung mass, and MUC2 was also positive in the left lung mass. Therefore, ampullary carcinoma was excluded and duodenal carcinoma was considered. Endoscopy also revealed a mass in the lateral wall of the descending duodenum. Immunohistochemistry of different tumors is shown in **Table 3**.

**Table 3:** Immunohistochemistry of different tumors.

	Immunohistochemistry	References
Primary adenocarcinoma of lung	TTF-1+, CK7+, CK20-	Su et al.(2006) (15)
Lung adenocarcinoma of digestive tract origin	TTF-1(-), CK7(-), CK20(+)	Su et al.(2006) (15)
Ampullary carcinoma	CK17(+), MUC4(+), MUC1(+)	Bakshi et al.(2019) (16)
Duodenal carcinoma	CK20(+), MUC2(+), CDX2(+)	Bakshi et al.(2019) (16)
Lymphoma	CD20+, CD3+, et al.	Higgins et al. (2008) (17)

Lymphoma can present with extranodal involvement (18). Invasion of the lungs then may cause cough, difficulty breathing, and other symptoms (19). Invasion of the digestive tract will cause gastrointestinal mass, abdominal pain, and other symptoms (20). When the skin is invaded, skin lesions occur (21). Our case had multiple lymph node enlargement, skin lesions, and pulmonary and duodenal masses. Therefore, lymphoma needs to be considered. Sarcoidosis is a noncaseating necrotizing granulomatous disease that can involve every organ, often involving the lungs, bilateral hilar lymph nodes, skin, and other organs (22). The patient had a left pulmonary mass, bilateral hilar lymphadenopathy, and rash. Therefore, sarcoidosis is also under consideration. The diagnosis of lymphoma and sarcoidosis is based on biopsy, and in addition to morphology, there are also typical immunophenotypes and molecular lesions to aid the diagnosis. Judging from the results of the biopsy, the diagnosis of lymphoma and sarcoidosis can be ruled out in our case.

The detection of tumor markers in the blood has certain reference values for tumor screening and diagnosis. Tumor markers in the gastrointestinal tract mainly include CEA, CA19-9, CA125, and CA72-4, of which CEA has the highest sensitivity (23–26), and tumor markers in lung cancer include CEA, CYFRA21-1, SCC, and NSE (27). In our case, tumor markers were elevated in both the gastrointestinal tract and the lung, indicating that the tumor markers were nonspecific and cannot be relied on alone to determine the origin of the primary tumor.

In addition, supraclavicular lymph node metastasis also plays a role in distinguishing the origin of the tumor. Metastasis of the left supraclavicular lymph node (Virchow lymph node) is associated with abdominal tumors, whereas the right supraclavicular lymph node is generally considered to be of thoracic origin (28). In this case, the left supraclavicular lymph node metastasis was found, which also confirmed that the primary lesion was in the gastrointestinal tract to a certain extent, and excluded the diagnosis of lung cancer.

The only possible cure for duodenal cancer is radical resection (29). Pancreaticoduodenectomy is considered the primary treatment option for eligible patients who can undergo radical tumor resection, while for palliative surgery, gastrointestinal and biliary bypass surgery is most commonly performed (7, 29). In our case, the cancer was advanced and incurable, so palliative bypass surgery was chosen.

The pathogenesis of small bowel cancer appears to be similar to that of colorectal cancer (30), and the treatment of small bowel adenocarcinoma has historically been based on the treatment strategies of colorectal cancer (5). In duodenal cancer, fluorouracil plus oxaliplatin is the most commonly used systemic chemotherapy regimen, with response rates ranging from 34% to 42%, median progression-free survival ranging from 6.9 to 8.2 months, and median overall survival ranging from 17.8 to 22.2 months. Irinotecan, cisplatin, and gemcitabine have also been reported. However, the response rate, median progression-free survival, and median overall survival were all relatively low (11). However, signet ring cell carcinoma can reduce the sensitivity to chemotherapy, and there is no literature to show the effect of systemic chemotherapy on duodenal signet ring cell carcinoma. The progression-free survival of our case was only 2 months, confirming to some extent the high malignancy and low sensitivity to chemotherapy of signet ring cell carcinoma. Previous studies did not recommend Her-2 and Ras gene testing in patients with duodenal cancer but did recommend testing for microsatellite instability (MSI) and mismatch repair (MMR) proteins and pembrolizumab monotherapy for MSI-high and Mismatch repair deficiency (dMMR) patients with duodenal cancer (11).

## Conclusion

There are several important points in the diagnostic process in this case that should be noted. First, although abdominal pain is the most common symptom of duodenal adenocarcinoma, when metastatic disease develops, the first symptom may be caused by metastatic disease. Secondly, when considering a pulmonary malignant tumor, it is necessary to determine whether the tumor is primary or secondary. Finally, poorly cohesive duodenal carcinoma mixed with signet ring cell carcinoma is very rare, and it is easy to be misdiagnosed when the tumor is metastatic. The diagnosis often requires a comprehensive combination of endoscopy, imaging, pathology, immunohistochemistry, tumor markers, and even the location of supraclavicular lymph nodes.

## Data availability statement

The original contributions presented in the study are included in the article/supplementary material. Further inquiries can be directed to the corresponding author.

## Ethics statement

Written informed consent was obtained from the individual for the publication of any potentially identifiable images or data included in this article.

## Author contributions

ST and XL were the patient’s physicians and collected the patient’s data. ST reviewed the literature and completed the first draft of the article. AW reviewed the article and made revisions. All authors contributed to the article and approved the submitted version.
